# “CATAStrophy,” a Genome-Informed Trophic Classification of Filamentous Plant Pathogens – How Many Different Types of Filamentous Plant Pathogens Are There?

**DOI:** 10.3389/fmicb.2019.03088

**Published:** 2020-01-21

**Authors:** James K. Hane, Jonathan Paxman, Darcy A. B. Jones, Richard P. Oliver, Pierre de Wit

**Affiliations:** ^1^Centre for Crop and Disease Management, School of Molecular and Life Sciences, Curtin University, Perth, WA, Australia; ^2^Curtin Institute for Computation, Faculty of Science and Engineering, Curtin University, Perth, WA, Australia; ^3^Department of Mechanical Engineering, Curtin University, Perth, WA, Australia; ^4^Laboratory of Phytopathology, Department of Plant Sciences, Wageningen University & Research, Wageningen, Netherlands

**Keywords:** fungi, plant pathogen, biotroph, necrotroph, hemibiotroph, CAZymes, metabolism

## Abstract

The traditional classification of fungal and oomycete phytopathogens into three classes – biotrophs, hemibiotrophs, or necrotrophs – is unsustainable. This study highlights multiple phytopathogen species for which these labels have been inappropriately applied. We propose a novel and reproducible classification based solely on genome-derived analysis of carbohydrate-active enzyme (CAZyme) gene content called CAZyme-Assisted Training And Sorting of -trophy (CATAStrophy). CATAStrophy defines four major divisions for species associated with living plants. These are monomertrophs (Mo) (corresponding to biotrophs), polymertrophs (P) (corresponding to necrotrophs), mesotrophs (Me) (corresponding to hemibiotrophs), and vasculartrophs (including species commonly described as wilts, rots, or anthracnoses). The Mo class encompasses symbiont, haustorial, and non-haustorial species. Me are divided into the subclasses intracellular and extracellular Me, and the P into broad and narrow host sub-classes. This gives a total of seven discrete plant-pathogenic classes. The classification provides insight into the properties of these species and offers a facile route to develop control measures for newly recognized diseases. Software for CATAStrophy is available online at https://github.com/ccdmb/catastrophy. We present the CATAStrophy method for the prediction of trophic phenotypes based on CAZyme gene content, as a complementary method to the traditional tripartite “biotroph–hemibiotroph–necrotroph” classifications that may encourage renewed investigation and revision within the fungal biology community.

## Introduction

Fungal and oomycete plant pathogens cause crop losses of ∼15–25% of yield potential (Fisher et al., 2018; Savary et al., 2019) and just five diseases destroy crops that could feed >600 million people (Fisher et al., 2012; Bebber and Gurr, 2015; Gurr et al., 2015). Combating such diseases is an ongoing challenge requiring good understanding of interactions between pathogens and hosts. Fungal and oomycete pathogens have been classified by modes of nutrition for over 130 years (de Bary and Garnsey, 1887), but in the last 50 years the dominant model has been a division into three “trophic” classes, biotrophs, hemibiotrophs, and necrotrophs (Thrower, 1966; Lewis, 1973). Non-pathogen species are described as symbionts (or commensals) when living on or within a living host without causing significant damage, or as saprotrophs (S) (or in older literature as saprophytes) when they extract nutrients solely from decaying biomaterials. The suffix “-trophic” emphasizes that this model refers to the feeding mode of the pathogens. Biotrophs feed on living host tissues and necrotrophs on dead tissues. Hemibiotrophs start infection as a biotroph and subsequently switch to necrotrophy [see Box 1 for a conventional statement of the definitions]. The biotrophic, hemibiotrophic, and necrotrophic classes have become associated with a number of other properties (Table 1).

**BOX 1. Conventional terms for describing plant pathogen trophic phenotypes.**

Biotroph – feeding from within living host cells throughout its lifecycle. Necrotroph – feeding from dead (or dying) host cells. Hemibiotroph – initially feeding as a biotroph and then switching to necrotrophy. Saprotroph – a fungus that only lives on dead organic material.

**TABLE 1:** Alleged typical properties of pathogenic trophic classes.

**Property**	**Biotroph**	**Hemibiotroph**	**Necrotroph**
Feeding (Scott, 1972; Parbery, 1996)	On living host cells	Initially on living and later on dying/dead host cells	On dead or dying host cells
Obligate or facultative (Scott, 1972; Parbery, 1996)	Obligate	Facultative	Facultative
Feeding structures (Gay, 1984; Mendgen et al., 2000; Laluk and Mengiste, 2010)	Haustoria	Haustorium-like structures (appressoria/hyphopodia) in some cases	No haustoria
Host range (Lewis, 1973; Lucas, 1998; Zeilinger et al., 2016)	Narrow	Narrow	Broad
Hormones involved in defense (Hammond-Kosack and Parker, 2003; Glazebrook, 2005)	Salicylic acid	Salicylic/Jasmonic acid	Jasmonic acid
Effectors (Stergiopoulos and de Wit, 2009; Tan et al., 2010; Koeck et al., 2011)	Avirulence effectors; gene-for-gene interactions	Avirulence effectors; gene-for-gene interactions	Host-specific toxins; necrotrophic effectors
Resistance genes (Glazebrook, 2005; Wang et al., 2014)	Qualitative	Qualitative	Quantitative

It is widely acknowledged that this model of plant pathogen classification leaves much to be desired. Many pathogens are placed by different authors in two and, in a few cases, all three classes (Oliver and Ip-Cho, 2004; Stotz et al., 2014). None of the features listed in Table 1 are diagnostic, with the possible exception that all obligate pathogens are biotrophic, but the converse is not true. There are substantial differences in the hemibiotrophic lifestyle with some species having a clear temporal division between biotrophic and necrotrophic phase, while in others the trophic phase can coincide in time but in differentiated tissues of the infected host. Classifications based on host-range or type of defense mechanism are not supported by well-established data. Furthermore nearly all resistance genes are, in some circumstances, quantitative (Poland et al., 2009).

The fundamental basis of the difference between biotrophy and necrotrophy – feeding on living and dead cells – is difficult to apply. Firstly, it is unclear precisely when a host cell dies and secondly, as all fungi and oomycetes feed by extracellular osmotrophic adsorption (Richards and Talbot, 2013), it is unclear which host cells can be said to be feeding the pathogen. Infected tissue might contain both living and dead host cells, both of which are releasing nutrients. Other groupings of plant pathogens have been proposed. For example, wilt pathogens are defined as colonizers of xylem vessels and surrounding parenchyma tissues and cause characteristic symptoms associated with water stress. It is unclear whether these pathogens have more in common with biotrophs or necrotrophs (Klosterman et al., 2011).

The first completed genome sequence was brewer’s yeast in 1996 (Goffeau et al., 1996) and fungal plant pathogen genomes followed from 2005. In this report, we studied 158 plant pathogen genomes including those of 143 fungal and 15 oomycete species or isolates (Pedro et al., 2016; Supplementary Data Sheet S1). The motivation was to determine whether an unbiased examination of this wealth of genome sequence data would reveal an objective and robust classification system that had predictive power. We sought a method that would exclusively utilize genome-derived sequences and not require expression analyses or any other *in vivo* assessments to predict the trophic phenotype of a novel pathogen species.

**FIGURE 1:**
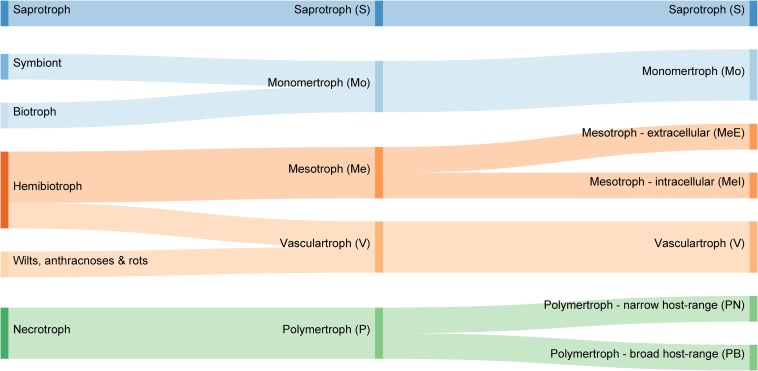
Comparison of common trophic terms used in plant pathology literature **(left)**, with our proposed novel classification system of five major trophic classes **(middle)** and nine sub-classes **(right)**.

In this study, we used counts of carbohydrate-active enzyme (CAZyme)-encoding genes (Lombard et al., 2014) to generate a novel classification of plant pathogens. Our analysis suggests the existing tripartite trophic classification system is unsustainable, highlights longstanding anomalies, and permits the objective prediction of trophic phenotype based on data common to all genome projects. The process grouped species with similar trophic phenotypes regardless of their phylogenetic history. We identified novel groups comprising four major plant pathogen classes [monomertrophs (Mo), polymertrophs (P), mesotrophs (Me), and vasculartrophs (V)], two of which could be further divided into two sub-classes (Figure 1). The Mo primarily metabolize simple sugars, P metabolize complex sugars, and Me have characteristics of both. These novel classes are roughly analogous to biotrophs, necrotrophs, and hemibiotrophs, respectively. The data included in this study were used to develop and train a predictive tool for CAZyme-Assisted Training And Sorting of -trophy (CATAStrophy), available online at https://github.com/ccdmb/catastrophy. We present the CATAStrophy method for the prediction of trophic phenotypes basedn on CAZyme gene content, as a complementary method to the traditional tripartite “biotroph–hemibiotroph–necrotroph” classifications that may encourage renewed investigation and revision within the fungal biology community.

## Results

Our goal was to use only genome sequences to determine whether existing or new classifications of filamentous plant pathogens were objectively supported, as gene transcript data or cell-biological observations would eliminate the universality of the approach. Initial investigations revealed that a small set of gene functions was necessary to reduce noise. We focused on genes encoding CAZymes (Cantarel et al., 2009; Lombard et al., 2014), a ubiquitous, large, and well-defined set that can be auto-annotated in a consistent manner. Furthermore, CAZyme genes typically reside in genome regions less prone to *de novo* assembly errors (Soanes et al., 2008). The CAZyme gene contents of 133 fungal and 15 oomycete species/*formae speciales*, and CAZyme annotations were assigned for 136–1314 genes in fungi and 255–793 genes in oomycetes (Supplementary Data Sheet S2).

**FIGURE 2:**
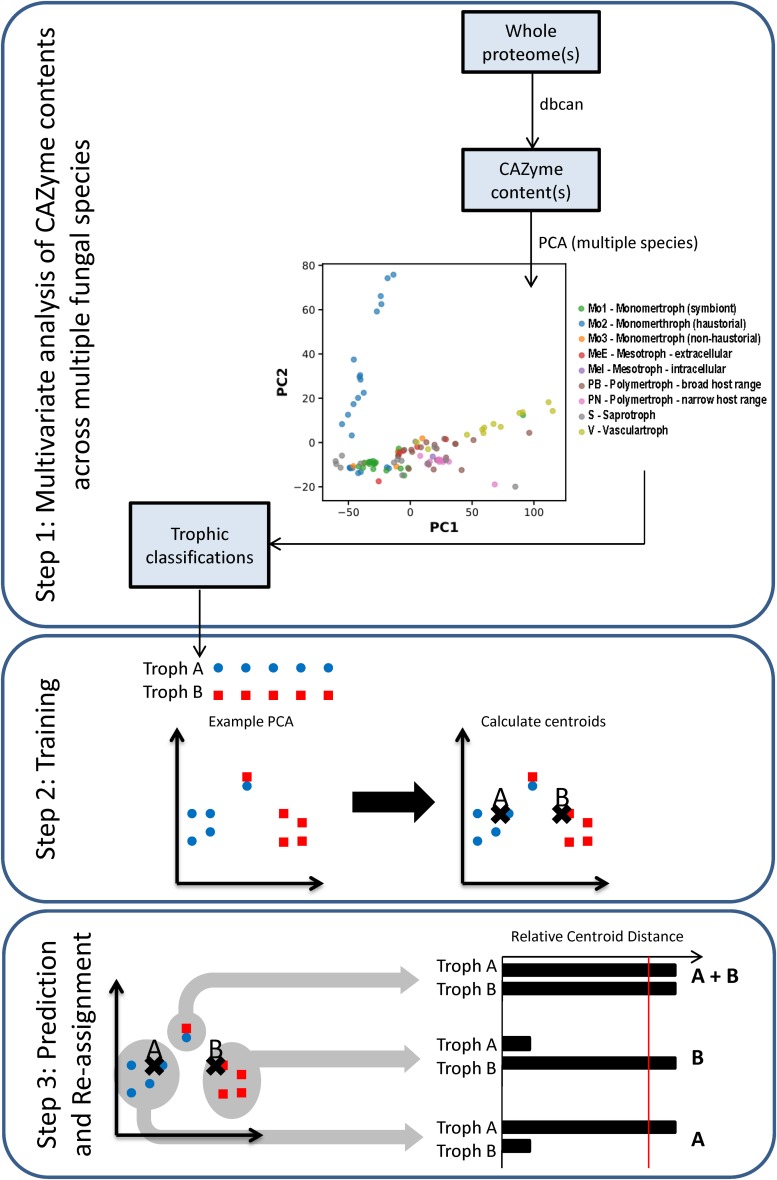
Workflow of the trophic prediction method. Step 1: CAZyme gene contents are compared across species using multivariate analysis. Step 2: trophic classifications are assigned to each species (Supplementary Data Sheet S1) and centroids are calculated for each trophic class. Step 3: relative centroid distances (RCDs) are calculated for each species, with the closest centroid assigned an RCD score of 1, the furthest as 0, and other centroid distances expressed as a relative proportion. Species were predicted as members of a major trophic class where RCD = 1 and assigned additional affinities for other classes or sub-classes where RCD ≥ 0.95. Importantly, species may be predicted after RCD calculation into a different class than was initially assigned.

**FIGURE 3:**
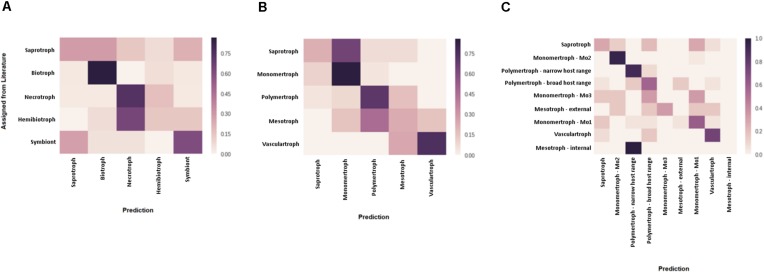
Assessment of predicted CATAStrophy classifications of fungal trophic phenotypes. Confusion matrices were used to assess the relative suitability of each nomenclature [literature-derived terms **(A)** compared, novel major classes **(B)**, and novel sub-classes **(C)**] for use in the CATAStrophy RCD method. CATAStrophy predictions were assessed for accuracy compared to the assigned terms listed in Supplementary Data Sheet S1.

Principal component analysis (PCA) of CAZyme contents across a training set of 85 fungal and oomycete species (Supplementary Data Sheet S1) allowed the separation of most of the species with the first two principal components (PCs) (Figure 2, Step 1), containing 56.5 and 10.7% of variation, respectively. PC2 separated species predominantly based on phylogeny, with the Oomycota generally having high values, Ascomycota low values, and Basidiomycota low to intermediate values. PC1 separated trophic classes into an approximate spectrum progressing from the traditionally classified S to biotrophs, hemibiotrophs, and necrotrophs. While a trend was apparent, using the trophic terms assigned based on commonly usage in literature (Figure 3 and Supplementary Data Sheet S1), these terms were not consistently clustered within the same regions of PCA space. We also used novel trophic classifications proposed in this study consisting of five major classes (Figure 2, Step 1), two of which were each sub-dividable into two sub-classes. Species commonly described as wilts formed a distinct group with high PC1 values and low PC2 values (Figure 2, Step 1), suggesting the need for the creation of a new class.

We propose a novel trophic nomenclature that contains five major classes (Figure 1, section “Materials and Methods”) and introduces new class names derived from our CAZyme-based approach. The S class remains unchanged, while the traditional biotroph and necrotroph classes are replaced by Mo and P, respectively, reflecting a preference for either monomeric or oligomeric/polymeric primary nutrient sources. Two novel classes are proposed which broadly replace the hemibiotrophs; these are Me (from “meso” meaning intermediate) and V, which comprises pathogens commonly described as wilts, anthracnoses, and rots. The P are divided into two sub-classes that correspond to polyphagy [broad host range (PB) or host-specificity (narrow host range {PN}). The Me class divided into two sub-classes corresponding to intracellular (MeI)] or extracellular (MeE) interactions. Hence, there are a total of four major classes of fungi and oomycetes that all interact with living plants (Mo, P, Me, and V) alongside the non-pathogenic S, and four informative sub-classes (MeI, MeE, PN, and PB).

After applying our novel nomenclatures to the PCA data (Figure 2, Step 1) we observed improvements in how species of the same trophic classification grouped into homogeneous clusters (Figures 3B,C). Our method for testing and predicting trophic phenotypes had to deal with cases where species were roughly equidistant to two or more clusters within the PCA space (Figure 2, Step 2). We therefore calculated centroids in the PCA space and developed metrics for the relative distances to the centroids of each trophic class, which we refer to herein as “relative centroid distance” (RCD) (Figure 2, Step 3).

We predicted each species as a member of one of the five major classes (S, Mo, Me, P, and V), and also assigned one or more secondary “affinities,” for sub-classes of the Me and P classes (MeI, MeE, PN, and PB) or alternate major classes that differed from the primary class prediction. We observed the RCD method (see the section “Materials and Methods”) using our novel trophic classes to be generally consistent with our overall biological expectations of trophic phenotypes (Figures 3B,C) and report our predictions for the 158 isolates included in this study (Table 2 and Supplementary Data Sheet S1). We observed several examples of distantly related taxa being predicted in the same trophic class and conversely species of the same genus accurately placed into different trophic classes. Rate of successful prediction (Supplementary Data Sheet S1) was 77% compared to terms derived from common usage in the literature; however, the curated success rate was 90% after taking into account recent literature revisions and other caveats outlined in the discussion and noted in Supplementary Data Sheet S1.

**TABLE 2:** Summary of predicted CATAStrophy classifications for selected fungal and oomycete species (full version in Supplementary Data Sheet S1).

**Species**	**Strain/isolate**	**Phylum/sub-** **phylum (-mycota)**	**Class** **(-mycetes)**	**Common literature-** **based description** **(-troph)**	**Assigned sub-** **class for training** **(pre-prediction)**	**Saprotroph**	**Monomertroph**	**Mesotroph –** **inttracellular**	**Mesotroph –** **extracellular**	**Polymertroph –** **narrow host range**	**Polymertroph –** **broad host range**	**Vasculartroph**	**Predicted** **major class**	**Predicted** **sub-class** **affinities**
*Alternaria alternata*	ATCC66891	Asco	Dothideo	Necro-	U	0.37	0.28	**0.98**	0.51	**1**	0.71	0.68	P	MeI, PN
*Alternaria brassicicola*	BMP1950	Asco	Dothideo	Necro-	PN	0.75	0.67	0.52	**0.97**	**1**	**0.97**	0	P	MeE, PN/PB
*Ascochyta rabiei*	ArDii	Asco	Dothideo	Necro-	U	0.60	0.51	0.46	0.93	0.85	**1**	0	P	PB
*Cochliobolus heterostrophus*	C5	Asco	Dothideo	Necro-	PN	0.37	0.31	0.73	0.51	**1**	0.65	0.37	P	PN
*Cochliobolus sativus* (syn. *Bipolaris sorokiniana*)	ND90Pr	Asco	Dothideo	Necro-	PN	0.41	0.35	0.64	0.57	**1**	0.68	0.22	P	PN
*Dothistroma septosporum*	NZE10	Asco	Dothideo	Hemibio-	MeE	0.89	0.98	0.26	**1**	0.57	0.76	0	Me	Mo, MeE
*Fulvia fulva* (syn *Cladosporium fulvum; Passalora fulva*)	CBS131901	Asco	Dothideo	Hemibio-	Mo	0.67	0.74	0.32	**1**	0.68	0.91	0	Me	MeE
*Leptosphaeria maculans*	v23.1.3	Asco	Dothideo	Hemibio-	MeE	**1**	0.93	0.47	**1**	0.68	**1**	0	S/Me/P	S, MeE, PB
*Parastagonospora nodorum*	SN15	Asco	Dothideo	Necro-	PN	0.41	0.32	0.59	0.56	**1**	0.67	0.24	P	PN
*Pseudocercospora fijiensis* (syn. *Mycosphaerella fijiensis*)	CIRAD86	Asco	Dothideo	Hemibio-	MeE	0.73	0.83	0.26	**1**	0.65	0.82	0	Me	MeE
*Pyrenophora teres* f. *teres*	0-1	Asco	Dothideo	Necro-	PN	0.47	0.4	0.48	0.67	**1**	0.75	0.05	P	PN
*Pyrenophora tritici-repentis*	Pt-1C-BFP	Asco	Dothideo	Necro-	PN	0.53	0.44	0.47	0.73	**1**	0.81	0	P	PN
*Ramularia collo-cygni*	DK05 Rcc001	Asco	Dothideo	Hemibio-	U	0.79	0.87	0.28	**1**	0.62	0.83	0	Me	MeE
*Venturia inaequalis*	20141010	Asco	Dothideo	Hemibio-	U	0.71	0.68	0.41	**1**	0.84	**1**	0	Me/P	MeE, PB
*Venturia pirina*	20150407	Asco	Dothideo	Hemibio-	U	0.74	0.75	0.44	**1**	0.84	**0.98**	0	Me	MeE, PB
*Zymoseptoria tritici*	IPO323	Asco	Dothideo	Hemibio-	MeE	0.86	0.97	0.24	**1**	0.57	0.73	0	Me	Mo, MeE
*Blumeria graminis*	DH14	Asco	Leotio	Bio-	Mo	0.90	**1**	0.21	0.81	0.44	0.60	0	Mo	–
*Botrytis cinerea*	B05	Asco	Leotio	Necro-	PB	0.61	0.5	0.40	0.75	0.52	**1**	0	P	PB
*Erysiphe necator*	C	Asco	Leotio	Bio-	Mo	0.90	**1**	0.22	0.81	0.45	0.60	0	Mo	–
*Hymenoscyphus fraxineus* (syn. *Chalara fraxinea*)	KW1	Asco	Leotio	Necro-	U	0.47	0.38	0.73	0.63	**1**	0.86	0.28	P	PN
*Sclerotinia borealis*	F-4128	Asco	Leotio	Necro-	PB	0.74	0.61	0.39	0.82	0.58	**1**	0	P	PB
*Sclerotinia sclerotiorum*	1980 UF-70	Asco	Leotio	Necro-	PB	0.91	0.82	0.39	0.94	0.59	**1**	0	P	PB
*Colletotrichum gloeosporioides*	Cg-14	Asco	Sordario	Hemibio-	MeI	0.27	0.19	0.90	0.37	0.65	0.55	**1**	V	–
*Colletotrichum graminicola*	M1.001	Asco	Sordario	Hemibio-	MeI	0.52	0.38	**1**	0.64	**1**	0.91	0.43	Me/P	MeI, PN
*Colletotrichum higginsianum*	IMI349063	Asco	Sordario	Hemibio-	MeI	0.39	0.3	**1**	0.52	0.75	0.75	0.54	Me	MeI
*Epichloë festucae*	E2368	Asco	Sordario	Symbiont	Mo	0.84	1	0.21	0.78	0.42	0.58	0	Mo	Mo
*Epichloë glyceriae*	E277	Asco	Sordario	Symbiont	Mo	0.84	1	0.30	0.93	0.56	0.69	0	Mo	Mo
*Fusarium graminearum*	PH-1	Asco	Sordario	Hemibio-	MeE	0.58	0.5	**0.97**	0.81	**0.98**	**1**	0.77	P	MeI, PN/PB
*Fusarium oxysporum* f. sp. *lycopersici*	4287	Asco	Sordario	Wilt	V	0.24	0.19	0.69	0.33	0.51	0.46	**1**	V	–
*Fusarium solani*	mpVI	Asco	Sordario	Wilt	V	0.24	0.18	0.69	0.33	0.50	0.45	**1**	V	–
*Gaeumannomyces graminis*	R3-111a-1	Asco	Sordario	Root	PB	0.65	0.56	0.74	0.75	**1**	0.88	0.04	P	PN
*Magnaporthe oryzae*	70-15	Asco	Sordario	Hemibio-	MeI	0.56	0.48	0.73	0.71	**1**	0.83	0.15	P	PN
*Magnaporthe poae*	ATCC64411	Asco	Sordario	Root	Mo	**0.99**	1	0.44	**0.95**	0.71	0.82	0	Mo	S, MeE
*Verticillium albo-atrum*	VaMs.102	Asco	Sordario	Necro-	V	0.84	0.72	0.58	0.94	0.77	**1**	0	P	PB
*Verticillium dahliae*	VdSo316	Asco	Sordario	Hemibio-	V	0.67	0.48	0.70	0.79	0.85	**1**	0	P	PB
*Rhizoctonia solani*	AG1-IA	Basidio	Agarico	Necro-	PB	0.57	0.38	0.71	0.67	0.65	**1**	0.06	P	PB
*Rhizoctonia solani*	AG8 WAC10335	Basidio	Agarico	Necro-	PB	**1**	0.93	0.47	**1**	0.68	**1**	0	S/Me/P	S, MeE, PB
*Melampsora laricis-populina*	98AG31	Basidio	Puccinio	Bio-	Mo	**0.98**	**1**	0.33	**0.99**	0.59	0.79	0	Mo	S, MeE
*Puccinia graminis*	UG99	Basidio	Puccinio	Bio-	Mo	**0.97**	**1**	0.32	**0.97**	0.59	0.78	0	Mo	S, MeE
*Puccinia striiformis*	PST-130	Basidio	Puccinio	Bio-	Mo	0.92	**1**	0.27	0.86	0.51	0.67	0	Mo	–
*Ustilago hordei*	Uh4857_4	Basidio	Ustilagino	Bio-	Mo	0.90	**1**	0.21	0.82	0.43	0.61	0	Mo	–
*Ustilago maydis*	521	Basidio	Ustilagino	Bio-	Mo	0.75	**1**	0.22	0.78	0.45	0.64	0	Mo	–
*Albugo candida*	ASM107853v1	Oo	Oo	Bio-	Mo	0.74	**1**	0.19	0.66	0.37	0.51	0	Mo	–
*Albugo laibachiic*	ENA1	Oo	Oo	Bio-	Mo	0.84	**1**	0.23	0.72	0.42	0.56	0	Mo	–
*Hyaloperonospora arabidopsidis*	Emoy2	Oo	Oo	Bio-	Mo	0.71	**1**	0.18	0.64	0.36	0.49	0	Mo	–
*Phytophthora ramorum*	CDFA1418886	Oo	Oo	Bio-	Mo	0.58	**1**	0.21	0.60	0.33	0.51	0	Mo	–
*Phytophthora sojae*	P6497	Oo	Oo	Bio-	Mo	0.51	**1**	0.24	0.58	0.31	0.53	0	Mo	–

*Relative centroid distance (RCD) scores from 0 to 1 are presented for each of the nine trophic sub-classes. An RCD value of 1 (bold and underlined) indicates membership in a major trophic class and a value ≥0.95 (bold) predicts affinity for one or more trophic sub-classes. Predicted trophic class and sub-classes are summarized in the right-hand columns. S, saprotroph; Mo, monomertroph; Me, mesotroph; MeI, mesotroph – intracellular; MeE, mesotroph – extracellular; P, polymertroph; PB, polymertroph – broad host range; PN, polymertroph – narrow host range; V, vasculartroph; U, unclassified (not included in training).*

## Discussion

### The Five-Trophic Classes: Saprotrophs, Monomertrophs, Polymertrophs, Mesotrophs, and Vasculartrophs

Since the inception of plant pathology, classification of filamentous fungal and oomycete plant pathogens into subgroups has been attempted based on nutritive phenotypes (de Bary and Garnsey, 1887). A tripartite division into biotrophs, necrotrophs, or hemibiotrophs has dominated the field for 50 years (Thrower, 1966; Lewis, 1973). It is striking that even with advancements in microscopy, allowing observations of host–microbe interactions at the cellular level, these divisions have persisted despite many obvious anomalies (Kuo et al., 2014; Stotz et al., 2014; Sánchez-Vallet et al., 2015; Videira et al., 2017). These divisions have been causally linked to broader features of their host interactions (Glazebrook, 2005) and thence directed strategies for disease control (Oliver, 2009; Burdon et al., 2014).

The genomics era has given us a plethora of data with which to generate an objective classification system that would aid development of sustainable control strategies for both familiar and emergent plant pathogens (Fisher et al., 2012). The CATAStrophy method provides a non-biased way to predict the trophic (sub-)class of filamentous plant pathogens solely based on their CAZyme gene content. The discussion below focuses on key species – we invite readers to view comprehensive reports of species and their trophic predictions in Supplementary Data Sheet S1 and Supplementary Text S1.

### Monomertrophs

Perhaps the most distinctive of the traditionally defined pathogens classes are the biotrophs. Archetypal biotrophs complete their lifecycles only on their specific hosts and typically exhibit clear-cut gene-for-gene host interactions involving biotrophic effectors (syn. avirulence determinants) (Tanaka et al., 2015). Their extreme host specialization is linked to the absence of several primary biosynthetic pathways (Supplementary Text S1). Archetypal biotrophs feed via specific structures, haustoria, which invaginate the host cell membranes and permit the adsorption of nutrients directly from the host cytoplasm (Staples, 2001). Haustoria have evolved multiple times and are found in Ascomycota (powdery mildews), Basidiomycota (rusts), and the Oomycota (downy mildews and *Phytophthora* species). They are also found in true symbionts, including the mycorrhizal Glomeromycota.

The CATAStrophy method linked phylogenetically disparate groups of traditional biotrophs and symbionts into the Mo class, including oomycetes (e.g., *Phytophthora* spp., *Albugo* spp., and *Hyaloperonospora arabidopsidis*), rust and smut fungi (e.g., *Puccinia* spp., *Ustilago* spp., and *Melampsora laricis-populina*), the powdery mildews (e.g., *Erysiphe necator* and *Blumeria graminis*), and known symbionts and mycorrhiza (e.g., *Epichloë* spp., *Pisolithus* spp., *Laccaria bicolour*, and *Tuber melanosporum*). Excluded from this class were traditionally defined biotrophs such as *Fulvia fulva* (syn. *Cladosporium fulvum*, *Passalora fulva*) and *Venturia* spp., which lack haustoria. Indeed, *F. fulva* was long regarded as a model for the biotrophs (de Wit, 2016). However, recent studies have concluded that both *F. fulva* and *Venturia* spp. are hemibiotrophic (Stotz et al., 2014).

The Mo class was the least well-predicted by CATAStrophy, in that haustorial and non-haustorial sub-classes could not be adequately distinguished, nor could the symbionts. Biotrophs and symbionts have low CAZYme (Supplementary Data Sheet S2) and secondary metabolite gene contents (Supplementary Text S1). This is consistent with a common strategy of causing minimal damage to host cells, i.e., producing fewer PAMPs or DAMPs. Free-living yeast species were also cryptically predicted in this class, likely due to their preference for unpolymerized sugars (Rodrigues et al., 2006) that parallel haustorial biotrophic metabolism (Hahn and Mendgen, 1997, 2001; Voegele et al., 2001). Yeasts and species like *N. crassa* are the first colonizers of rich sources of sugars and amino acids, and some strains lack enzymes needed even for modestly polymerized substrates (e.g., sucrose). Species in the Mo class generally have the lowest number of CAZymes, consistent with this explanation (Hahn and Mendgen, 2001). An improved method that might be able to resolve these issues, such as through use of and expanded set of appropriate functional annotations, may be possible to address in a follow-up study.

### Polymertrophs

Methods to classify facultative plant pathogens are less widely accepted. The term necrotroph has been applied to pathogens that cause rapid necrosis when inoculated onto hosts and whose culture filtrates also cause necrosis when applied to host tissue (Solomon et al., 2006). CATAStrophy grouped genera or species already widely accepted as necrotrophic into the P class, including: *Alternaria* spp., *Botrytis cinerea* (syn. *Botryotinia fuckeliana*), *Cochliobolus* (syn. *Bipolaris*) spp., *Pyrenophora* spp., *Parastagonospora nodorum*, *Ascochyta rabiei*, *Rhizoctonia solani*, *Gaeumannomyces graminis*, and *Sclerotinia* spp. *Fusarium graminearum* is commonly reported as a hemibiotroph, but polymertrophy is consistent with its broad host range and reliance on mycotoxins. *Verticillium* spp. were predicted as P despite initial assignment as V prior to RCD prediction (see below). *Magnaporthe oryzae* was also predicted as a P, and although commonly described as hemibiotrophic, it is capable of causing rapid necrosis. In contrast, the closely related *M. poae* was predicted as a Mo consistent with it known properties.

#### Broad Host-Range Polymertrophs

*Botrytis cinerea* quintessentially represents this sub-class. Others included *Sclerotinia* spp., *Verticillium* spp., *Aspergillus* spp., *Alternaria brassicicola*, *A. rabiei*, and *F. graminearum*. *R. solani* is divided into sexually incompatible anastomosis groups (AGs) exhibiting variable breadths in host ranges. The AG1-IA isolate (infecting rice) was predicted as PB but the AG8 isolate (infecting multiple legume and cereal species) was predicted across the S, Me(MeE), and PB classes. Both *R. solani* AG8 and *Leptosphaeria maculans* were predicted across three primary classes (S/Me/P, with affinities for MeE and PB sub-classes). Both exhibit wide host-ranges and complex and elongated life cycles that may indicate prolonged saprotrophic or biotrophic phases prior to necrotrophy.

#### Narrow Host-Range Polymertrophs

Broad host-range polymertrophs and PN pathogens can be distinguished by CAZyme content (Choquer et al., 2007; Andrew et al., 2012; Baroncelli et al., 2016), the former having expanded CAZyme contents ensuring activity across multiple hosts (Baroncelli et al., 2016), which may permit reduced reliance on effectors. Conversely, PN pathogens require less CAZyme diversity relative to the PB sub-class and are commonly reported to use host-specific necrotrophic effectors (Stergiopoulos and de Wit, 2009). The PN sub-class conformed well to conventional expectations, and included *Pyrenophora* spp., *P. nodorum*, *Cochliobolus* spp., and *Alternaria* spp. (except *Alt. brassicicola*) (see also Supplementary Text S1).

### Mesotrophs

Hemibiotrophs are the most problematic traditional classification and some species described in this division were not predicted as Me. Instead our analysis grouped facultative biotrophic species that have longer latent periods than necrotrophs and do not use toxins as a primary virulence determinant into the Me class. They include most (but not all) *Colletotrichum* spp., *Venturia* spp., *Zymoseptoria* spp., *F. poae*, *Pseudocercospora fijiensis*, *F. fulva*, *L. maculans*, and *R. solani* AG8. Our analysis supported a further division into two sub-classes similar to that proposed earlier (Perfect et al., 1999) based on invasion of either intracellular or extracellular host tissues.

#### Extracellular (Non-appressorial) Mesotrophs

Hemibiotrophs including *L. maculans*, *Zymoseptoria* spp., and *P. fijiensis* exhibit an elongated latent phase prior to necrotrophy and were appropriately predicted with MeE affinity. *Venturia* spp. and *C. fulvum* were also predicted as MeE, in agreement with their recent re-classifications as hemibiotrophs (Stotz et al., 2014). *C. fulvum* – long regarded as a model biotroph – grows biotrophically under controlled greenhouse conditions with optimal temperature and relative humidity (de Wit, 2016), but under variable conditions or natural infection can cause noticeable necrosis.

#### Intracellular (Appressorial) Mesotrophs

The MeI sub-class was initially assigned to species possessing appressoria-like feeding structures formed on the host surface prior to host penetration, exemplified by the *Colletotrichum* spp. Almost all *Colletotrichum* spp. were predicted as MeI, with the exception of *C. gloeosporioides* (V). Other appressorial species including *M. oryzae*, *G. graminis*, and *Alternaria* spp. were predicted instead as P (excepting *A. longipes*, MeI). *F. poae* and *F. graminearum* (P) were predicted with MeI affinity, which is supported in the latter by reports of mycotoxin-producing appressorium-like structures. While this class was initially assigned to appressorial hemibiotroph species prior to RCD prediction, the MeI sub-class appears not to be strictly linked to the presence of appressoria but still correlates to intracellular host interactions. This mirrors how reports of appressoria do not align consistently with the intracellular hemibiotrophic phenotype.

### Vasculartrophs

We propose a novel V class which contains pathogens that are associated with wilt, anthracnose, and rot symptoms and grouped separately from the Mo, Me, or P classes. Several “wilt-like” species are not well-defined in terms of their mode of nutrition, but our analysis suggests that V are most similar in CAZyme content to the PB sub-class. This V class was initially assigned to the *Fusarium* spp. (excluding *F. graminearum*) and *Verticillium* spp. prior to RCD prediction. In final trophic predictions (Step 3) however, *Verticillium* spp., *F. poae*, and *F. lansethiae* were not predicted in this class. *Verticillium* spp. and *Fusarium* spp., despite both being commonly referred to as “wilts,” do exhibit several differences including: host-range (*Verticillium* is broader), climate preference (*Verticillium* prefers cooler temperatures), and severity with less vascular browning and no cell death in *Verticillium* but more browning and necrosis in *Fusarium* wilt on tomato. Thus, the prediction of *Verticillium* outside this group (PB) may be due to genuine biological features that need to be further investigated. Although the *Colletotrichum* spp. are predominantly predicted as mesotrophic, *C. gloeosporioides*, *C. simmondsii*, and *C. nymphaeae* were predicted as primarily vasculartrophic.

## Conclusion

The long history of the biotroph–hemibiotroph–necrotroph classification of plant pathogens (de Bary and Garnsey, 1887) is evidenced by its persistence in major textbooks and reviews (Horbach et al., 2011). Despite its ubiquity, the tripartite classification has long been regarded as problematic (Oliver and Ip-Cho, 2004; Glazebrook, 2005; Kuo et al., 2014; Stotz et al., 2014; Sánchez-Vallet et al., 2015; Videira et al., 2017). Increased availability of genomic data has allowed us to re-examine the suitability of this nomenclature. The CATAStrophy method allows for the prediction of trophic classes based solely on CAZYme gene content. In place of the three major classes of pathogen, we propose four novel pathogen classes: Mo, P, Me, and V.

Carbohydrate-active enzyme-Assisted Training And Sorting of -trophy focuses attention on the properties linking and separating these groups and provides a basis for a reproducible, objective, and unbiased classification of fungal trophic phenotypes. Current trends in whole-genome sequencing techniques and costs have led to a rapid increase in the number of fungal species sequenced. Correspondingly, the species studied by these techniques have rapidly spread from a few species with historically high economic and scientific relevance to species with local or recent impact. A good example is ash-dieback and Ramularia leaf spot (Saunders et al., 2014; Stam et al., 2018). There are clear differences in the strategies adopted to combat haustorial biotrophic and narrow-host range necrotrophic plant pathogens (Oliver, 2009; Burdon et al., 2014). Thus, the economic and societal impact of a rapid assessment of the causal organism of a novel disease could be significant. As microbial genomics data grow in volume, we anticipate an emerging need for bioinformatic techniques such as CATAStrophy that can predict agriculturally relevant phenotypes from genomic data, particularly as only a minor fraction of plant pathogenic fungi have been studied in detail. The CATAStrophy method suggests a novel and more detailed grouping of pathogens which we hope will stimulate the development and testing of hypotheses relating to pathogenicity, virulence, and control measures.

## Materials and Methods

### Prediction of Carbohydrate-Active Enzyme Contents

Whole proteome (i.e., predicted gene translations) sequences were obtained in FASTA format as per Supplementary Data Sheet S1 and Supplementary Text S1. The CAZyme (Cantarel et al., 2009; Lombard et al., 2014) functional annotations were utilized to represent *a priori* evidence reporting the “trophic type.” CAZyme classes were annotated for all species via HMMER 3.0 (as per dbCAN recommendations, i.e., hmmscan with the -domtblout parameter, then dbcan hmmscan-parser. sh with 80 aa minimum alignment length, *e*-value < 1*e*−5 and >30% coverage of HMM) (Eddy, 2010) and the dbCAN (version 6) set of CAZyme HMMs (Yin et al., 2012), listed in full in Supplementary Data Sheet S2.

### Organization of Reported Trophic Phenotypes Into Discrete Classes

We tested three discrete nomenclatures that describe the trophic phenotype. The first trophic nomenclature was assigned to species based on the terms – S, symbiont, biotroph, hemibiotroph, and necrotroph – commonly reported in published literature (Table 2 and Figure 3A). The second nomenclature uses five major divisions (S, Mo, Me, P, and V) (Figure 3B). Nomenclature 3 uses the five major divisions (S, Mo, Me, P, and V), and included sub-divisions for MeI, MeE, PN, PB and three sub-divisions of the Mo (symbionts, haustorial, and non-haustorial; Figure 1 top panel) that were later obsoleted (Table 2 and Figure 3C). Due to difficulties in resolving the sub-classes within the Mo, we assigned them numerical labels (Mo1, Mo2, and Mo3, respectively) where they appear in Supplementary Material (Supplementary Data Sheet S1), but for the purpose of summarizing CATAStrophy predictions have merged them into a single Mo class (Mo). Importantly, all three nomenclatures were initially based on reports derived from peer-reviewed literature (Supplementary Data Sheet S1). The three nomenclatures were tested for their relative efficacy (Figure 3) and nomenclature 2 (S, Mo, Me, P, V) is the primary one used for subsequent analyses presented in this study.

### Prediction of Trophic Classes via Multivariate Analysis

The number of genes in each species assigned to each CAZyme class was used in PCA using singular value decomposition via scikit-learn v 0.18.1 (Pedregosa et al., 2011) to cluster species (Figure 2). Species were each assigned a trophic class based on the most commonly used term derived from literature reports, or the equivalent term from our novel proposed nomenclatures. Centroids corresponding to each trophic sub-class were calculated based on the positions in PCA space of the species assigned that class (Supplementary Data Sheet S1). Each species was then unassigned from its designated trophic class, its position in PCA space relative to centroids was calculated, and a RCD score was calculated for each species to assess the relative likelihood of its membership in each class. Centroids were re-calculated for the assessment of each species during RCD analysis, with the species currently being assessed being removed from centroid calculations so as not to influence the prediction. The centroid closest to a species in PCA space was assigned an RCD score of 1, with other centroids expressed as a normalized proportion of the closest centroid distance. RCD scores were rounded to two decimal places. Using data based on initial manual assignment of the novel classes and sub-classes (Table 2 and Supplementary Data Sheet S1), species were predicted to belong to broad classes (Table 2) where RCD = 1 with high confidence, and also assigned additional “affinities” for sub-classes (Table 2) if RCD ≥ 0.95 at a lower confidence. RCD scores for the biotroph sub-divisions Mo1, Mo2, and Mo3 are reported individually in Supplementary Data Sheet S1, but only the maximum of these scores is reported for the Mo class in Table 2. Using this method it is possible for trophic classes to be revised, i.e., a species may be predicted in a different class than it was originally assigned to prior to RCD calculation. In order to demonstrate the efficacy of the newly proposed trophic nomenclatures for the CATAStrophy RCD method, each of the three nomenclatures (literature-derived, novel major classes, and novel sub-classes) was tested separately via the CATAStrophy method and the predictions were assessed using confusion matrices that the predictions to assigned terms (Figure 3). The PCA plot and principle component coordinates for each species included in the initial CATAStrophy analysis (i.e., not unassigned in Supplementary Data Sheet S1) are provided in Supplementary Data Sheet S3.

## Data Availability Statement

All datasets generated for this study are included in the article/Supplementary Material. CATAStrophy software available at https://github.com/ccdmb/catastrophy.

## Author Contributions

JH and DJ performed the bioinformatics analysis. DJ and JP performed the multivariate analysis. JH and RO wrote the manuscript. JH, RO, and PW edited the manuscript. All authors read and approved the manuscript.

## Conflict of Interest

The authors declare that the research was conducted in the absence of any commercial or financial relationships that could be construed as a potential conflict of interest.

## Supplementary Material

DATA SHEET S1 Full list of fungal and oomycete species and isolates used in this study, their assigned trophic classifications based on literature consensus, and their predicted CATAStrophy classifications.

DATA SHEET S2 CAZyme annotations assigned to species via dbCAN.

DATA SHEET S3 Plot and PCA coordinates for the first 16 principle components derived from CAZyme gene contents for 110 fungal and oomycete species and isolates used to build the CATAStrophy RCD predictive method.

TEXT S1 References for species and genome resources cited in this study, and additional discussion of CATAStrophy predictions for selected species.
