# Supplementary material for: “CATAStrophy,” a Genome-Informed Trophic Classification of Filamentous Plant Pathogens – How Many Different Types of Filamentous Plant Pathogens Are There?
Source: Front Microbiol. 2020 Jan 21;10:3088. doi: 10.3389/fmicb.2019.03088 (PMC6986263; doi:10.3389/fmicb.2019.03088)
Supplement: TEXT S1 — References for species and genome resources cited in this study, and additional discussion of CATAStrophy predictions for selected species. [file Table_1.DOCX]

**Species, in approximate order of appearance in main text:**

Oomycetes: *Phytophthora* spp., *Albugo* spp. and *Hyaloperonospoera arabidopsidis* (*1-5*)

Mildews, Rusts and Smuts: (*6-13*)

*Puccinia* spp. (*6, 7*)

*Ustilago* spp. (*12, 13*)

*Melampsora laricis-populina* (*7*)

*Erysiphe necator* (*14*)

*Blumeria graminis* (*15*)

*Epichloë* spp. (*16*)

*Pisolithus* spp. (*17*)

*Laccaria* *bicolor* (*18*)

*Tuber* *melanosporum* (*19*)

*Cladosporium fulvum* (syn. *Passalora fulva; Fulvia fulva*) (*20-23*)

*Venturia* spp (*24, 25*)

*Neurospora crassa* (*26, 27*)

*Alternaria* spp. (*28, 29*)

*Botrytis cinerea* (*30-32*)

*Cochliobolus* (syn. *Bipolaris*) spp. (*33-35*)

*Pyrenophora* spp. (*36-39*)

*Parastagonospora nodorum* (*40, 41*)

*Ascochyta rabiei* (*42*)

*Rhizoctonia solani* (*43-45*)

*Gaeumannomyces graminis* (*46, 47*)

*Sclerotinia* spp (*48, 49*)

*Fusarium* spp.: (*50*)

*Fusarium* *graminearum* (*51-56*)

*Fusarium poae* (*57*)

*Verticillium* spp. (*58*)

*Magnaporthe* spp.:

*Magnaporthe oryzae* (*59, 60*)

*Magnaporthe poae* (*61, 62*)

*Trichoderma virens* and *Trichoderma reesii* (*63*)

*Leptosphaeria maculans* (*64*)

*Trametes versicolor* & *Stereum hirsutum* (*65*)

*Colletotrichum* spp. (*66-74*)

*Zymoseptoria* spp*.* (*75, 76*)

*Pseudocercospora* (syn. *Mycosphaerella*) *fijiensis* (*77, 78*)

*Dothistroma septosporum* (*20*)

**Species in order of appearance in Table 2:**

| **Species** | **Strain**  **/Isolate** | **Ref(s)** |
| --- | --- | --- |
| *Alternaria alternata* | ATCC66891 | (*28, 29*) |
| *Alternaria brassicicola* | BMP1950 | (*28, 29*) |
| *Ascochyta rabiei* | ArDii | (*42*) |
| *Cochliobolus heterostrophus* | C5 | (*33-35*) |
| *Cochliobolus sativus* (syn*. Bipolaris sorokiniana*) | ND90Pr | (*33-35*) |
| *Dothistroma septosporum* | NZE10 | (*20*) |
| *Leptosphaeria maculans* | v23.1.3 | (*64*) |
| *Parastagonospora nodorum* | SN15 | (*40, 41*) |
| *Passalora fulva (*syn *Cladosporium fulvum; Fulvia fulva*) | CBS131901 | (*20-23*) |
| *Pseudocercospora fijiensis* (syn*. Mycosphaerella fijiensis*) | CIRAD86 | (*77, 78*) |
| *Pyrenophora teres teres* | 0-1 | (*36-39*) |
| *Pyrenophora tritici-repentis* | Pt-1C-BFP | (*36-39*) |
| *Ramularia collo-cygni* | DK05 Rcc001 | (*79-81*) |
| *Venturia inaequalis* | 20141010 | (*24, 25*) |
| *Venturia pirinia* | 20150407 | (*24, 25*) |
| *Zymoseptoria tritici* | IPO323 |  |
| *Blumeria graminis* | DH14 | (*15*) |
| *Erysiphe necator* | C | (*14*) |
| *Botrytis cinerea* | B05 | (*30-32*) |
| *Sclerotinia borealis* | F-4128 | (*48, 49*) |
| *Sclerotinia sclerotiorum* | 1980 UF-70 | (*48, 49*) |
| *Colletotrichum gloeosporioides* | Cg-14 | (*66-74*) |
| *Colletotrichum graminicola* | M1.001 | (*66-74*) |
| *Colletotrichum higginsianum* | IMI349063 | (*66-74*) |
| *Epichloë festucae* | E2368 | (*16*) |
| *Epichloë glyceriae* | E277 | (*16*) |
| *Fusarium graminearum* | PH-1 | (*51-56*) |
| *Fusarium oxysporum* f. sp*. lycopersici* | 4287 | (*50*) |
| *Fusarium solani* | mpVI | (*50*) |
| *Gaeumannomyces graminis* | R3-111a-1 | (*46, 47*) |
| *Magnaporthe oryzae* | 70-15 | (*59, 60*) |
| *Magnaporthe poae* | ATCC64411 | (*61, 62*) |
| *Verticillium albo-atrum* | VaMs.102 | (*58*) |
| *Verticillium dahliae* | VdSo316 | (*58*) |
| *Rhizoctonia solani* | AG1-IA | (*43-45*) |
| *Rhizoctonia solani* | AG8 WAC10335 | (*43-45*) |
| *Melampsora laricis-populina* | 98AG31 | (*7*) |
| *Puccinia graminis* | UG99 | (*6, 7*) |
| *Puccinia striformis* | PST-130 | (*6, 7*) |
| *Ustilago hordei* | Uh4857_4 | (*12, 13*) |
| *Ustilago maydis* | 521 | (*12, 13*) |
| *Albugo candida* | ASM107853v1 | (*1-5*) |
| *Albugo laibachii* | ENA1 | (*1-5*) |
| *Hyaloperonospora arabidopsidis* | Emoy2 | (*1-5*) |
| *Phytophthora ramorum* | CDFA1418886 | (*1-5*) |
| *Phytophthora sojae* | P6497 | (*1-5*) |

**Supplemental Results & Discussion**

This section contains notes and comments that are supplemental to the results and discussion sections in the main text:

*Neurospora crassa* (*82*) has long been regarded as a model saprotroph, but was recently also reported to be a pathogen of Scot’s pine (*83*), hence its prediction in the pathogenic monomertroph class is reasonable.

The role of *Phaeoacremonium aleophilum* in causing esca on grapevine has been scrutinised and its proposed role has ranged from pathogen to endophyte (*84*), however in this study it was predicted as an external mesotroph.

*Eutypa lata* (Dead arm of grapevine) (*85*) and *Rhytidhysteron rufulum* (citrus stem canker) (*86*) are predicted as narrow-host range polymertrophs.

*Trichoderma virens* - a mycoparasite and commercial biocontrol agent of soil-borne plant pathogenic fungi (e.g. *R. solani*, *S. sclerotiorum*, *P. ultimum* etc.)- is predicted in the polymertroph class and is distinct from its saprotrophic sister species *T. reeseii* (*87*).

The “turkey tail” and “false turkey tail” tree pathogens, *Trametes versicolor* and *Stereum hirsutum*, are usually described as saprotrophic (*65*) but were both predicted in the polymertroph class with PB sub-class affinity. This is more consistent with their known roles as broad host-range pathogens of trees.

*Alternaria brassisicola* was unique among its genus (cf. *A. alternata*) in having an affinity for the PB sub-class as opposed to the PN sub-class, which is consistent with observed differences in host range (*88*).

The sister species *Moniliophthora roreri* which causes frosty pod rot disease in cacao (*89, 90*) and *M. perniciosa* (*91*) are both commonly reported as hemibiotrophs (*91, 92*). *M. roreri* was predicted in this study as a vasculartroph which is consistent with its rot symptoms. *M. perniciosa* was predicted as a saptrotroph, which is a reasonable description of its observed lifestyle (saprotrophy and biotrophy), hence the hemibiotroph term has been inaccurately used for this species.

*Ramularia collo-cygni* (*79-81*) is commonly referred to as a necrotroph causing spot symptoms (ramularia leaf spot on barley), and in this study was predicted as a mesotroph. The spot lesions produced by this pathogen are distinct from most spot-causing necrotrophs as *R. collo-cygni* lesions are visible from both sides of the leaf surface (*79*). However these symptoms appear to be non-vascular in nature as its hyphae are observed to aggregate around vascular bundles, but do not penetrate them (*93*).

Narrow host-range polymertrophs/necrotrophs are commonly reported to rely on host-specific effector proteins, and there are several in this group which have documented histories of their recent acquisition. *Pyrenophora tritici-repentis* causes the disease tan spot which emerged as recently as 1941 and has since become one of the major pathogens of wheat worldwide (*38, 39*). The emergence of tan spot is strongly linked to the acquisition by lateral gene transfer of the necrotrophic effector *ToxA,* likely from *P. nodorum* (*38*). *Cochliobolus heterostrophus* (syn. *Bipolaris maydis*) has a similar pathogenic history to tan spot. Prior to 1962 *C. heterostrophus* race 0 was regarded as a mild pathogen of maize (*35*). However, 1962 saw a major epidemic of Southern Corn Leaf Blight, due to the emergence of Race T that carried the T-toxin effector. Race T was specifically virulent on maize carrying the CMS-T male sterility gene. Yield losses of 20% were reported in 1970s but when CMS-T maize lines were replaced, *C. heterostrophus* was controlled and has not been a major threat since (*34*). Whether the reliance on effectors and their potentially recent lateral gene transfer is related to the CAZyme content of a pathogen is unclear, however we speculate that a likely consequence of adaptation to a narrow host specialisation is a subsequent reduction in CAZyme diversity.

**References:**

1. B. J. Haas *et al.*, Genome sequence and analysis of the Irish potato famine pathogen *Phytophthora infestans*. *Nature* **461**, 393-398 (2009).

2. B. M. Tyler *et al.*, *Phytophthora* genome sequences uncover evolutionary origins and mechanisms of pathogenesis. *Science* **313**, 1261-1266 (2006).

3. M. G. Links *et al.*, *De novo* sequence assembly of *Albugo candida* reveals a small genome relative to other biotrophic oomycetes. *BMC Genomics* **12**, 503 (2011).

4. E. Kemen *et al.*, Gene gain and loss during evolution of obligate parasitism in the white rust pathogen of *Arabidopsis thaliana*. *PLoS Biol* **9**, e1001094 (2011).

5. L. Baxter *et al.*, Signatures of adaptation to obligate biotrophy in the *Hyaloperonospora arabidopsidis* genome. *Science* **330**, 1549-1551 (2010).

6. D. Cantu *et al.*, Next generation sequencing provides rapid access to the genome of *Puccinia striiformis* f. sp. *tritici*, the causal agent of wheat stripe rust. *PLoS One* **6**, e24230 (2011).

7. S. Duplessis *et al.*, Obligate biotrophy features unraveled by the genomic analysis of rust fungi. *Proc Natl Acad Sci U S A* **108**, 9166-9171 (2011).

8. A. L. Pendleton *et al.*, Duplications and losses in gene families of rust pathogens highlight putative effectors. *Front Plant Sci* **5**, 299 (2014).

9. P. D. Spanu, The genomics of obligate (and nonobligate) biotrophs. *Annu Rev Phytopathol* **50**, 91-109 (2012).

10. P. D. Spanu *et al.*, Genome expansion and gene loss in powdery mildew fungi reveal tradeoffs in extreme parasitism. *Science* **330**, 1543-1546 (2010).

11. Z. Zhao, H. Liu, C. Wang, J.-R. Xu, Comparative analysis of fungal genomes reveals different plant cell wall degrading capacity in fungi. *BMC genomics* **14**, 274 (2013).

12. M. Bölker, C. W. Basse, J. Schirawski, *Ustilago maydis* secondary metabolism—from genomics to biochemistry. *Fungal Genetics and Biology* **45**, S88-S93 (2008).

13. J. Kamper *et al.*, Insights from the genome of the biotrophic fungal plant pathogen *Ustilago maydis*. *Nature* **444**, 97-101 (2006).

14. L. Jones *et al.*, Adaptive genomic structural variation in the grape powdery mildew pathogen, *Erysiphe necator*. *BMC genomics* **15**, 1081 (2014).

15. L. Frantzeskakis *et al.*, Signatures of host specialization and a recent transposable element burst in the dynamic one-speed genome of the fungal barley powdery mildew pathogen. *BMC genomics* **19**, 381 (2018).

16. C. L. Schardl *et al.*, Plant-Symbiotic Fungi as Chemical Engineers: Multi-Genome Analysis of the *Clavicipitaceae* Reveals Dynamics of Alkaloid Loci. *Plos Genet* **9**, e1003323 (2013).

17. A. Kohler *et al.*, Convergent losses of decay mechanisms and rapid turnover of symbiosis genes in mycorrhizal mutualists. *Nat Genet* **47**, 410-415 (2015).

18. F. Martin *et al.*, The genome of *Laccaria bicolor* provides insights into mycorrhizal symbiosis. *Nature* **452**, 88-U87 (2008).

19. F. Martin *et al.*, Perigord black truffle genome uncovers evolutionary origins and mechanisms of symbiosis. *Nature* **464**, 1033-1038 (2010).

20. P. J. G.M. De Wit *et al.*, The genomes of the fungal plant pathogens *Cladosporium fulvum* and *Dothistroma septosporum* reveal adaptation to different hosts and lifestyles but also signatures of common ancestry. *Plos Genet* **8**, e1003088 (2012).

21. R. A. Ohm *et al.*, Diverse lifestyles and strategies of plant pathogenesis encoded in the genomes of eighteen Dothideomycetes fungi. *PLoS Pathog* **8**, e1003037 (2012).

22. S. Videira *et al.*, *Mycosphaerellaceae*–Chaos or clarity? *Studies in mycology* **87**, 257-421 (2017).

23. B. P. H. J. Thomma, H. P. Van Esse, P. W. Crous, P. J. G. M. De Wit, *Cladosporium fulvum* (syn. *Passalora fulva*), a highly specialized plant pathogen as a model for functional studies on plant pathogenic *Mycosphaerellaceae*. *Molecular Plant Pathology* **6**, 379-393 (2005).

24. J. K. Bowen *et al.*, *Venturia inaequalis*: the causal agent of apple scab. *Mol Plant Pathol* **12**, 105-122 (2011).

25. C. H. Deng *et al.*, Comparative analysis of the predicted secretomes of Rosaceae scab pathogens *Venturia inaequalis* and *V. pirina* reveals expanded effector families and putative determinants of host range. *BMC Genomics* **18**, 339 (2017).

26. J. E. Galagan *et al.*, The genome sequence of the filamentous fungus *Neurospora crassa*. *Nature* **422**, 859-868 (2003).

27. H. C. Kuo *et al.*, Secret lifestyles of *Neurospora crassa*. *Sci Rep* **4**, 5135 (2014).

28. H. X. Dang, B. Pryor, T. Peever, C. B. Lawrence, The *Alternaria* genomes database: a comprehensive resource for a fungal genus comprised of saprophytes, plant pathogens, and allergenic species. *BMC Genomics* **16**, 239 (2015).

29. B. P. H .J.Thomma, *Alternaria* spp.: from general saprophyte to specific parasite. *Mol Plant Pathol* **4**, 225-236 (2003).

30. J. A. Van Kan *et al.*, A gapless genome sequence of the fungus *Botrytis cinerea*. *Mol Plant Pathol* **18**, 75-89 (2017).

31. J. Amselem *et al.*, Genomic analysis of the necrotrophic fungal pathogens *Sclerotinia sclerotiorum* and *Botrytis cinerea*. *Plos Genet* **7**, e1002230 (2011).

32. B. Williamson, B. Tudzynski, P. Tudzynski, J. A. van Kan, *Botrytis cinerea*: the cause of grey mould disease. *Mol Plant Pathol* **8**, 561-580 (2007).

33. B. J. Condon *et al.*, Comparative genome structure, secondary metabolite, and effector coding capacity across *Cochliobolus* pathogens. *Plos Genet* **9**, e1003233 (2013).

34. B. A. Horwitz, B. J. Condon, B. G. Turgeon, in *Genomics of Soil-and Plant-Associated Fungi*. (Springer, 2013), pp. 213-228.

35. O. C. Yoder, *Cochliobolus heterostrophus, cause of southern corn leaf blight.*, (Academic Press, San Diego, 1988), vol. 6, pp. 93-112.

36. S. R. Ellwood *et al.*, A first genome assembly of the barley fungal pathogen *Pyrenophora teres* f. *teres*. *Genome Biol* **11**, R109 (2010).

37. V. A. Manning *et al.*, Comparative genomics of a plant-pathogenic fungus, *Pyrenophora tritici-repentis*, reveals transduplication and the impact of repeat elements on pathogenicity and population divergence. *G3 (Bethesda)* **3**, 41-63 (2013).

38. T. L. Friesen *et al.*, Emergence of a new disease as a result of interspecific virulence gene transfer. *Nature Genetics* **38**, 953-956 (2006).

39. R. P. Oliver, P. S. Solomon, New developments in pathogenicity and virulence of necrotrophs. *Current Opinion in Plant Biology* **13**, 415-419 (2010).

40. R. A. Syme *et al.*, Comprehensive Annotation of the *Parastagonospora nodorum* Reference Genome Using Next-Generation Genomics, Transcriptomics and Proteogenomics. *PLoS One* **11**, e0147221 (2016).

41. R. P. Oliver, T. L. Friesen, J. D. Faris, P. S. Solomon, *Stagonospora nodorum*: from pathology to genomics and host resistance. *Annu Rev Phytopathol* **50**, 23-43 (2012).

42. S. Verma *et al.*, Draft genome sequencing and secretome analysis of fungal phytopathogen *Ascochyta rabiei* provides insight into the necrotrophic effector repertoire. *Sci Rep-Uk* **6**, 24638 (2016).

43. J. K. Hane, J. P. Anderson, A. H. Williams, J. Sperschneider, K. B. Singh, Genome sequencing and comparative genomics of the broad host-range pathogen *Rhizoctonia solani* AG8. *Plos Genet* **10**, e1004281 (2014).

44. A. Zheng *et al.*, The evolution and pathogenic mechanisms of the rice sheath blight pathogen. *Nature Communications* **4**, 1424 (2013).

45. B. Sneh, L. Burpee, A. Ogoshi, *Identification of Rhizoctonia species*. (APS press, 1991).

46. J. Freeman, E. Ward, *Gaeumannomyces graminis*, the take-all fungus and its relatives. *Mol Plant Pathol* **5**, 235-252 (2004).

47. L. H. Okagaki *et al.*, Genome sequences of three phytopathogenic species of the Magnaporthaceae family of fungi. *G3: Genes, Genomes, Genetics* **5**, 2539-2545 (2015).

48. M. Derbyshire *et al.*, The Complete Genome Sequence of the Phytopathogenic Fungus *Sclerotinia sclerotiorum* Reveals Insights into the Genome Architecture of Broad Host Range Pathogens. *Genome Biol Evol* **9**, 593-618 (2017).

49. A. V. Mardanov, A. V. Beletsky, V. V. Kadnikov, A. N. Ignatov, N. V. Ravin, Draft Genome Sequence of *Sclerotinia borealis*, a Psychrophilic Plant Pathogenic Fungus. *Genome Announc* **2**, e01175-01113 (2014).

50. L. J. Ma *et al.*, *Fusarium* pathogenomics. *Annu Rev Microbiol* **67**, 399-416 (2013).

51. L. J. Harris, M. Balcerzak, A. Johnston, D. Schneiderman, T. Ouellet, Host-preferential *Fusarium graminearum* gene expression during infection of wheat, barley, and maize. *Fungal Biol* **120**, 111-123 (2016).

52. R. S. Goswami, H. C. Kistler, Heading for disaster: *Fusarium graminearum* on cereal crops. *Mol Plant Pathol* **5**, 515-525 (2004).

53. L. Guo, L.-J. Ma, in *Genomics of Plant-Associated Fungi: Monocot Pathogens*. (Springer, 2014), pp. 103-122.

54. K. Kazan, D. M. Gardiner, J. M. Manners, On the trail of a cereal killer: recent advances in *Fusarium graminearum* pathogenomics and host resistance. *Mol Plant Pathol* **13**, 399-413 (2012).

55. X. W. Niu, Z. Y. Zheng, Y. G. Feng, W. Z. Guo, X. Y. Wang, The *Fusarium graminearum* virulence factor FGL targets an FKBP12 immunophilin of wheat. *Gene* **525**, 77-83 (2013).

56. M. J. Boenisch, W. Schafer, *Fusarium graminearum* forms mycotoxin producing infection structures on wheat. *BMC Plant Biol* **11**, 110 (2011).

57. A. Vanheule *et al.*, Living apart together: crosstalk between the core and supernumerary genomes in a fungal plant pathogen. *BMC Genomics* **17**, 670 (2016).

58. S. J. Klosterman *et al.*, Comparative Genomics Yields Insights into Niche Adaptation of Plant Vascular Wilt Pathogens. *Plos Pathogens* **7**, e1002137 (2011).

59. R. A. Dean *et al.*, The genome sequence of the rice blast fungus *Magnaporthe grisea*. *Nature* **434**, 980-986 (2005).

60. R. A. Wilson, N. J. Talbot, Under pressure: investigating the biology of plant infection by *Magnaporthe oryzae*. *Nat Rev Microbiol* **7**, 185-195 (2009).

61. P. J. Landschoot, N. Jackson, *Magnaporthe-Poae* Sp-Nov, a Hyphopodiate Fungus with a Phialophora Anamorph from Grass Roots in the United-States. *Mycol Res* **93**, 59-62 (1989).

62. L. H. Okagaki *et al.*, Genome Sequences of Three Phytopathogenic Species of the Magnaporthaceae Family of Fungi. *G3 (Bethesda)* **5**, 2539-2545 (2015).

63. D. Martinez *et al.*, Genome sequencing and analysis of the biomass-degrading fungus *Trichoderma reesei* (syn. *Hypocrea jecorina*). *Nat Biotechnol* **26**, 553-560 (2008).

64. T. Rouxel *et al.*, Effector diversification within compartments of the *Leptosphaeria maculans* genome affected by Repeat-Induced Point mutations. *Nature Communications* **2**, 202 (2011).

65. D. Floudas *et al.*, The Paleozoic origin of enzymatic lignin decomposition reconstructed from 31 fungal genomes. *Science* **336**, 1715-1719 (2012).

66. R. Baroncelli *et al.*, Gene family expansions and contractions are associated with host range in plant pathogens of the genus *Colletotrichum*. *BMC Genomics* **17**, 555 (2016).

67. J. Crouch *et al.*, in *Genomics of Plant-Associated Fungi: Monocot Pathogens*. (Springer, 2014), pp. 69-102.

68. P. Gan *et al.*, Comparative genomic and transcriptomic analyses reveal the hemibiotrophic stage shift of *Colletotrichum* fungi. *New Phytol* **197**, 1236-1249 (2013).

69. R. J. O'Connell *et al.*, Lifestyle transitions in plant pathogenic *Colletotrichum* fungi deciphered by genome and transcriptome analyses. *Nat Genet* **44**, 1060-1065 (2012).

70. S. E. Perfect, H. B. Hughes, R. J. O'Connell, J. R. Green, *Colletotrichum*: a model genus for studies on pathology and fungal–plant interactions. *Fungal Genetics and Biology* **27**, 186-198 (1999).

71. N. O. Ogbebor, A. T. Adekunle, D. A. Enobakhare, Inhibition of *Colletotrichum gloeosporioides* (Penz) Sac. causal organism of rubber (*Hevea brasiliensis* Muell. Arg.) leaf spot using plant extracts. *Afr J Biotechnol* **6**, 213-218 (2007).

72. C. Sangeetha, R. Rawal, Nutritional studies of *Colletotrichum gloeosporioides* (Penz.) Penz. and Sacc. the incitant of mango anthracnose. *World Journal of Agricultural Sciences* **4**, 717-720 (2008).

73. U. Damm *et al.*, The *Colletotrichum orbiculare* species complex: Important pathogens of field crops and weeds. *Fungal Divers* **61**, 29-59 (2013).

74. C. Sherriff *et al.*, Ribosomal DNA-Sequence Analysis Reveals New Species Groupings in the Genus *Colletotrichum*. *Exp Mycol* **18**, 121-138 (1994).

75. S. B. Goodwin *et al.*, Finished genome of the fungal wheat pathogen *Mycosphaerella graminicola* reveals dispensome structure, chromosome plasticity, and stealth pathogenesis. *Plos Genet* **7**, e1002070 (2011).

76. J. Grandaubert, A. Bhattacharyya, E. H. Stukenbrock, RNA-seq-Based Gene Annotation and Comparative Genomics of Four Fungal Grass Pathogens in the Genus *Zymoseptoria* Identify Novel Orphan Genes and Species-Specific Invasions of Transposable Elements. *G3 (Bethesda)* **5**, 1323-1333 (2015).

77. R. E. A. Isaza *et al.*, Combating a Global Threat to a Clonal Crop: Banana Black Sigatoka Pathogen *Pseudocercospora fijiensis* (Synonym *Mycosphaerella fijiensis*) Genomes Reveal Clues for Disease Control. *Plos Genet* **12**, e1005876 (2016).

78. G. H. J. Kema, D. Z. Yu, F. H. J. Rijkenberg, M. W. Shaw, R. P. Baayen, Histology of the pathogenesis of *Mycosphaerella graminicola* in wheat. *Phytopathology* **86**, 777-786 (1996).

79. G. R. D. McGrann *et al.*, The genome of the emerging barley pathogen *Ramularia collo-cygni.* *BMC Genomics* **17**, 584 (2016).

80. R. Stam *et al.*, The evolutionary history of the current global *Ramularia collo-cygni* epidemic. *bioRxiv*, 215418 (2017).

81. R. Stam *et al.*, A New Reference Genome Shows the One-Speed Genome Structure of the Barley Pathogen *Ramularia collo-cygni.* *Genome Biol Evol* **10**, 3243-3249 (2018).

82. J. E. Galagan *et al.*, The genome sequence of the filamentous fungus *Neurospora crassa. Nature* **422**, 859 (2003).

83. H.-C. Kuo *et al.*, Secret lifestyles of Neurospora crassa. *Scientific reports* **4**, (2014).

84. V. Hofstetter *et al.*, What if esca disease of grapevine were not a fungal disease? *Fungal Divers* **54**, 51-67 (2012).

85. B. Blanco-Ulate, P. E. Rolshausen, D. Cantu, Draft genome sequence of the grapevine dieback fungus *Eutypa lata* UCR-EL1. *Genome announcements* **1**, e00228-00213 (2013).

86. R. A. Ohm *et al.*, Diverse lifestyles and strategies of plant pathogenesis encoded in the genomes of eighteen Dothideomycetes fungi. *PLoS Pathogens* **8**, e1003037 (2012).

87. D. Martinez *et al.*, Genome sequencing and analysis of the biomass-degrading fungus *Trichoderma reesei* (syn. *Hypocrea jecorina*). *Nature biotechnology* **26**, 553 (2008).

88. Y. Cho, How the Necrotrophic Fungus *Alternaria brassicicola* Kills Plant Cells Remains an Enigma. *Eukaryotic Cell* **14**, 335-344 (2015).

89. H. Evans, Pod rot of cacao caused by *Moniliophthora* (*Monilia*) *roreri*. *Phytopathological papers*, (1981).

90. R. H. Fulton, The *Cacao* Disease Trilogy - Black Pod, Monilia Pod Rot, and Witches-Broom. *Plant Dis* **73**, 601-603 (1989).

91. J. M. C. Mondego *et al.*, A genome survey of *Moniliophthora perniciosa* gives new insights into Witches' Broom Disease of cacao. *BMC Genomics* **9**, 548 (2008).

92. L. W. Meinhardt *et al.*, *Moniliophthora perniciosa*, the causal agent of witches’ broom disease of cacao: what's new from this old foe? *Molecular Plant Pathology* **9**, 577-588 (2008).

93. M. Kaczmarek *et al.*, Infection strategy of Ramularia collo-cygni and development of ramularia leaf spot on barley and alternative graminaceous hosts. *Plant Pathology* **66**, 45-55 (2017).
